# Round the Hospitals

**Published:** 1917-05-26

**Authors:** 


### ROUND THE HOSPITALS.

A DELIGHTFUL instance of the gracious thoroughness with which Queen Alexandra does everything she undertakes was supplied by her visit to the London Hospital, of which she is President, on the 21st instant to present the Royal Red Cross to Miss Luckes, the matron. The arrangements were admirable, for the ceremony took place in one of the summer-houses in the Nurses' Garden—the Garden of Eden—in the presence of all the sisters and nurses who could be spared from the wards, who saluted the Royal party as they entered by raising the right hand. After the presentation Her Majesty opened the Queen Alexandra Nursing Home, which has been established for sick and ailing nurses, and went through it that she might satisfy herself that nothing was wanting that could add to their comfort. Queen Alexandra also spoke to many of the patients who were in the garden on stretchers and wheeled chairs. She showed particular interest in the garden, and complimented the lady gardener on the condition in which the grounds were kept.

LAST week we referred at length to the terms of the Central Committee's latest attempt to mislead and betray British nurses into a false position, and have since received written evidence that some nurses, as we expected, added their names to the petition which accompanied the manifesto, in the belief that it was in support of the College of Nursing. Nurses who have made this mistake have written to withdraw their names from the Central Committee's petition to the Prime Minister, and have expressed justifiable indignation at the action of this Committee. We are awaiting the action of the College Council in regard to this manifesto, and we hope the College will address themselves not only to the members on the Register, but to the nurses' homes, associations, clubs, co-operations and generally throughout the country. It is of the first importance that misleading statements issued for a purpose should be promptly dealt with and disposed of on the facts.

THE report of the Committee appointed by the Army Council to inquire into the resources of the country in trained nurses and women partly trained in nursing, so as to enable it to suggest the most economical method of utilising their services for civil and military purposes, has been printed. It has not been generally circulated, but copies are announced to be obtainable from the Secretary of the War Office, though we have failed to receive one. The *Times* of the 23rd instant, page 3, contains a summary of some of the recommendations of the Committee's Report, which is dated, by the way, January 24, 1917, that is, before the entry of the United States into the war.

THE late Archdeacon Wilberforce was very human, and had great personal influence for good over those he taught and worked amongst. He appealed strongly to nurses, and did them continuous good. Very naturally, many nurses visited Westminster Abbey on the 13th instant, and were rejoiced to see the Archdeacon's grave had been decorated with a profusion of white lilies, crimson carnations, roses, and lilies of the valley on the anniversary of his death. The brilliant colouring of the flowers and the glorious sunshine made the visitors rejoice, for Archdeacon Wilberforce's teaching and help had cheered many a tired nurse and strengthened her to face with new enthusiasm the battle of life. The Archdeacon was well known at most of our London hospitals, and once on a visit to a patient he said to a sister, "I firmly believe hospital sisters and nurses are doing real angels' work." He always saw good in everything and everybody, having supreme faith in the belief that there was sterling worth at the bottom in the majority of men and women. He had a special love for animals, and especially for the dog. Once in a discussion on the question of whether dogs had souls, he asked, "And why not? They are sometimes infinitely better than their owners." The nurses do well to bear their friend the Archdeacon in ever-tender memory.

MISS AGNES BAGNALL, the matron of the Royal Southern Hospital, Liverpool, has kindly sent us a report of the work in her department during 1916. The nursing staff now numbers eighty, a larger number than in 1915 owing to an increase in the beds. Of twenty probationers entered for training three left at the end of their trial month, and two subsequently without qualifying for a certificate. Of the remaining fifteen nurses who received certificates, six left for military work, three were appointed sisters in the hospital, two entered for maternity training, one for district work, two for private nursing, and the last of the fifteen married. This hospital has a private staff of ten nurses, who take charge of the wards in the sisters' absence on holiday when not employed with private cases. Of the older members of the staff Miss Mackintosh, after fifteen years' service as a sister, has been appointed matron to the Royal Infirmary, Oldham. A large number of certificated nurses attached to this infirmary are absent on military work, some of whom have appeared in the Honours Lists. Miss L. E. Jolly, matron, serving in France since the outbreak of war, has been awarded the Royal Red Cross. The same honour has been conferred upon Sister Mary Edwards and Nurse Mary Clark, who are both serving in France. Sister B. E. Barrett, who went to Salonika in June 1916, has had the Croix de Guerre awarded to her by the French for bravery during an air raid, and Nurses Casserley and Cullwick have both been mentioned in dispatches. At the Royal Southern Hospital, Liverpool, under Miss Bagnall, economy is studied and practised. It is an excellent rule, too, that nurses must be well fed in order to do their best work, and the supply of butter has been continued, a privilege much appreciated by the nurses.

ROUND THE HOSPITALS—(*continued*).

The field covered by women workers, ever extending, is now so wide as to give every woman with some ability an opportunity to pick and choose her sphere of work from a wide area. The outlook for municipal energies, as the centre to which all citizens are properly coming to look, where everything which affects the well-being, progress and development of the individual finds a focus, is momentous for good, and this field has already provided many openings for women workers. The same remark applies to the County Councils, whose sphere of usefulness cannot fail to be largely extended for good, owing to the awakening and development in agriculture and the general call for abundant and adequate, up-to-date house accommodation for workers on the land. Durham County Council has fixed the salary of the superintendent health visitor, Miss H. S. Cooper Hodgson, at £180, and the commencing salary of the health visitors at £105, rising to £135 as a maximum in three years.

Modesty is a virtue, rather than a gift, so far as human beings are concerned. We have all heard of hospital matrons who are too audacious in their many demands and who make their boards of management uneasy and suspicious with regard to the well-being of those who are working under their supervision. But, on the other hand, there are up and down the country quite a number of hospital matrons whose chief defect in their mental equipment is a rooted timidity. There is, for instance, the matron who knows perfectly well that she cannot keep her staff and cannot even get the right quality of nurse because the domestic accommodation offered is not what it should be. Such a matron has one clear duty before her: one thing she must do in justice to her hospital, in justice to her staff, and in justice to herself—she must persuade the governing body that a nurses' home is absolutely necessary. Yet the matron may be, alas! too timid to do more than throw out an occasional plaintive hint on the subject. She cannot summon the nerve to tackle the position fully and courageously for fear of jeopardising her own popularity with the governors. There is the matron, too, who fails to secure a remedy for the grave shortcomings of those with whom her own work is in close relation. Timidity must find no part in the work of a hospital matron, for so much depends upon her initiative and enterprise that to be influenced by it must ultimately prove fatal to her usefulness and success.

The reduction of bread-eating still holds the foremost place in institution economies. In one large V.A.D. hospital we hear that the matron has adopted the plan of dealing out a 2-lb. loaf every four days to each nurse, who keeps it in her own room and brings it in to each meal with her until it is finished, thus ensuring that she gets ½ lb. of bread a day and no more. We can hardly conceive of a more unsatisfactory method of rationing than this. It is the old allowance system in its worst possible form. It distinctly stimulates the appetite for bread since rigid limitation invariably has this effect. It makes no distinction of persons, and it condemns each nurse on the fourth day to the disagreeable task of consuming the stale heel of her loaf, the appearance of which has grown distasteful to her from much hoarding. It needs to be reiterated that in households where intelligent catering and cooking are carried out the war ration of bread is seldom, if ever, exceeded, while all have enough and to spare. The quantities of bread consumed per head in pre-war days in several hospitals where averages have been carefully computed did not reach the total of ½ lb. a day, and should more than this be consumed it exposes the fact that an insufficient supply of other articles has forced the diet down to a monotonous level prejudicial to health.

